# MxA for differentiating viral and bacterial infections in adults: a prospective, exploratory study

**DOI:** 10.1007/s15010-023-01986-0

**Published:** 2023-02-03

**Authors:** Matthäus Metz, Guido A. Gualdoni, Heide-Maria Winkler, Alexandra-Maria Warenits, Johannes Stöckl, Heinz Burgmann, Stefan Winkler, Zoe Anne Oesterreicher

**Affiliations:** 1 Division of Infectious Diseases and Tropical Medicine, Department of Medicine I, Medical University of Vienna, 1090 Vienna, Austria; 2 Division of Endocrinology and Metabolism, Department of Internal Medicine III, Medical University of Vienna, 1090 Vienna, Austria; 3 Division of Nephrology and Dialysis, Department of Medicine III, Medical University of Vienna, 1090 Vienna, Austria; 4 Department of Emergency Medicine, Medical University of Vienna, 1090 Vienna, Austria; 5 Institute of Immunology, Center of Pathophysiology, Infectiology, and Immunology, Medical University of Vienna, 1090 Vienna, Austria; 6 Internal Medicine 2, Gastroenterology and Hepatology and Rheumatology, University Hospital of St. Poelten, 3100 St. Poelten, Austria

**Keywords:** Viral infection, MxA, Mx1, Myxovirus resistance protein 1, Antibiotic resistance, Biomarker

## Abstract

**Purpose:**

Inappropriate antibiotic prescription in patients with viral infections contributes to the surge of antibiotic resistance. Viral infections induce the expression of the antiviral protein MxA in monocytes, which is a promising biomarker to differentiate between viral and bacterial diseases. In this prospective, exploratory study, we aimed to determine the diagnostic value of monocyte MxA expression in adults with viral, bacterial or co-infections.

**Methods:**

We measured monocyte MxA expression using flow cytometry in a cohort of 61 adults with various viral, bacterial and co-infections including patients receiving immunosuppressive therapy.

**Results:**

Monocyte MxA expression in virus-infected patients was significantly higher compared to bacterial infections (83.3 [66.8, 109.4] vs. 33.8 [29.3, 47.8] mean fluorescence intensity [MFI]; *p* < 0.0001) but not co-infections (53.1 [33.9, 88.9] MFI). At a threshold of 62.2 MFI, the area under the ROC curve (AUC) to differentiate between viral and bacterial infections was 0.9, with a sensitivity and specificity of 92.3% and 84.6%, respectively. Immunosuppressive therapy did not affect monocyte MxA expression in virus-infected patients.

**Conclusion:**

Our findings corroborate the diagnostic performance of MxA in differentiating viral and bacterial infections but also point to an important caveat of MxA in viral-bacterial co-infections. This study extends previous reports and indicates that MxA is also a useful biomarker in immunocompromised patients.

## Introduction

Prolonged duration of illness, high rates of mortality and increasing treatment costs are the consequences of infectious diseases with antibiotic resistant bacteria. Differentiation between viral and bacterial infections can be challenging and frequently results in inappropriate antibiotic prescription due to diagnostic uncertainty which further drives antimicrobial resistance [1, 2]. Thus, novel diagnostic methods that facilitate the diagnosis of viral infections are urgently needed to prevent a progression of the already drastic prevalence of antibiotic resistance [2]. While the routinely available biomarkers C-reactive protein (CRP) and procalcitonin (PCT) can assist clinicians in determining the etiology of an infectious disease, they lack sufficient specificity [3–5]. Direct pathogen detection, although often leading to diagnosis, also has some limitations including inaccessibility of infection site, fever without source, time to results, differentiation between disease causing pathogen or mere colonizer [6].

Providing a predominantly viral-induced biomarker in addition to routinely available biomarkers of infection and inflammation could improve diagnostic accuracy and thus prevent antibiotic overuse. Viral infections induce a cellular defense mechanism that includes the secretion of type I interferons [7]. These cytokines activate the transcription of a variety of antiviral proteins including myxovirus resistance protein A (MxA) [7]. Several pediatric studies indicate that MxA can facilitate the differentiation between viral and bacterial infections [8–10]. Especially, the combination of MxA and CRP, markers for viral infections and inflammation, respectively, offers promising results as a rapid point of care test in children and adults with respiratory infections [11–13]. The aim of this study was to evaluate MxA in routine practice in an adult population including immunocompromised patients with various bacterial and viral infections.

## Methods and materials

This prospective, exploratory study was conducted at the Department of Internal Medicine I, Division of Infectious Diseases and Tropical Medicine and at the Department of Emergency Medicine of the Medical University of Vienna between December 2018 and June 2019. Patients aged ≥ 18 with clinical suspicion of infectious disease or with confirmed infectious disease were included. Exclusion criteria were as follows: infectious disease within the last 4 weeks, interferon treatment, recent chemotherapy, recent treatment with biologicals, and vaccination within the last 4 weeks. Sample collection was performed as part of a routine blood draw.

### Group allocation

**Fig. 1:**
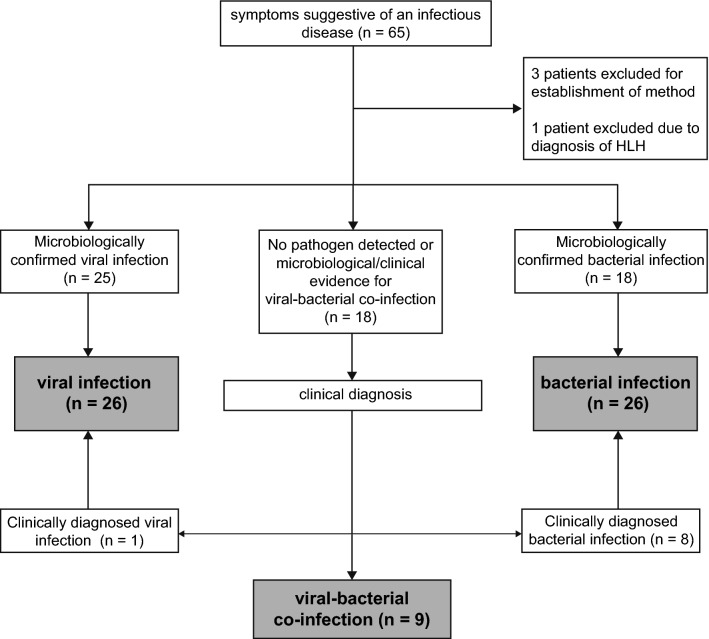
Diagnostic algorithm. Patients were allocated to the bacterial or viral group in case a pathogen that is compatible with patient’s symptoms was microbiologically identified or the diagnosis was made by the study team based on history, physical examination, radiological examination and laboratory results. In case a viral and bacterial pathogen was detected or the simultaneous infection was likely, patients were allocated to the viral-bacterial co-infection group. Samples from the first 3 patients were used to establish the FACS method. One patient was excluded due to hemophagocytic lymphohistiocytosis (HLH)

The participants were assigned to the following 5 groups depending on the routine diagnostic findings according to the algorithm in Fig. 1: confirmed viral infection, confirmed bacterial infection, clinically diagnosed viral infection, clinically diagnosed bacterial infection, bacterial-viral co-infection. A confirmed infection was defined as the identification of a pathogen compatible with the patient’s symptoms by routine microbiologic testing. Systematic, predefined testing for potential pathogens was not part of the study design and was decided by the treating physician depending on the clinical presentation of each included individual. Microbiological testing involved: blood cultures or bacterial cultures from, e.g., abscesses, pleural effusion or removed catheters, broad spectrum PCR from EDTA blood, PCR test from sputum on *Mycoplasma pneumoniae*, urine antigen test for *S. pneumoniae, H. influenzae, L. pneumophila*, urine test strips and urine cultures and PCR tests for influenzae virus and respiratory syncytial virus using nasopharyngeal swabs. In case of diarrhea, stool samples were obtained for bacterial cultures to detect growth of *Salmonella* spp., *Sighella* spp., *Campylobacter* spp, *Yersinia* spp, or for *C. difficile* toxin and antigen determination. PCRs for rotavirus, adenovirus, norovirus and enterovirus were obtained. In case of positive microbiological results after inclusion, participants were assigned to the respective group. In the case no pathogen was detected, the clinical diagnosis of infection was made based on history, clinical signs and symptoms and results of the routine diagnostic tests (C-reactive protein [CRP], procalcitonin and white blood cell count, urinary strips, chest X-ray or CT scans) performed at the discretion of the treating physician. An expert in infectious diseases reviewed the clinical diagnosis. All diagnoses were made without knowledge of MxA levels.

### Analytical procedures

**Fig. 2:**
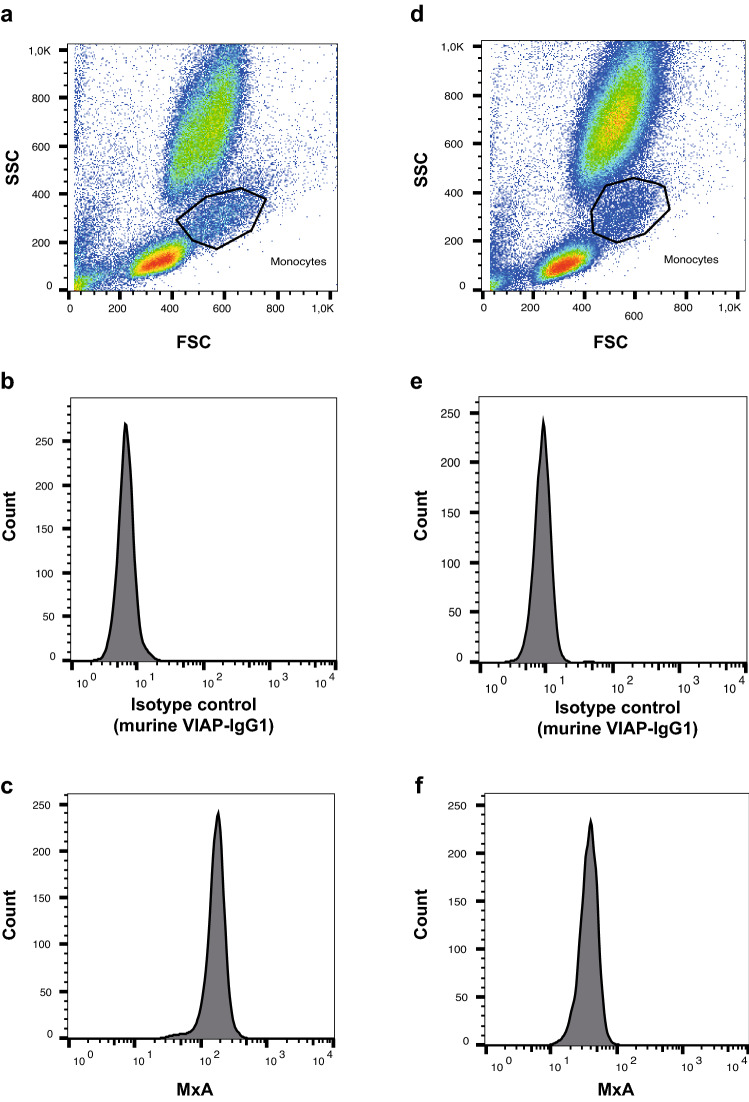
Flow cytometry analysis of monocyte MxA expression. Representative measurement of monocyte MxA expression in a patient with a viral infection (left column, **a-c**) and a patient with a bacterial infection (right column, **d-f**)

*Detection of monocyte MxA*. Fifty microliters of whole blood collected in lithium heparin tubes was diluted in 1 mL phosphate-buffered saline (PBS) and centrifuged for 5 min at 300 g. Supernatant was removed, and the procedure was repeated once. Cells in the remaining pellet were fixed and permeabilized using Fix&Perm (Nordic MUbio, Susteren, Netherlands) according to the manufacturer’s instructions. The cell pellet was reconstituted in 100µL fixation medium and incubated for 15 min at room temperature. Five milliliters PBS was added, and samples were centrifuged for 5 min at 300 g. Supernatant was removed, and the cell pellet was resuspended in 100µL permeabilization medium. Thereafter, 20µL of a saponin-buffered FITC-conjugated anti-MxA antibody (kindly provided by An der Grub BioResearch and validated previously [14]) was added; samples were vortexed and incubated for another 15 min at room temperature. Afterwards, cells were washed twice with PBS, centrifuged, resuspended in sheath fluid and analyzed immediately. Flow cytometry was performed using a BD FACS Calibur flow cytometer, and data were analyzed with FlowJo™ Software (BD Life Sciences, New Jersey, USA). Monocytes were identified using forward and side scatter, and the mean fluorescence intensity (MFI) was used as the main study parameter. Fluorescence signal of an isotype control (murine VIAP-IgG1, calf intestine alkaline phosphatase specific, generated and validated in our laboratory [14, 15]) was subtracted from the MxA MFI to account for unspecific binding on Fc receptors and monocyte auto-fluorescence (Fig. 2).

*Serum CRP levels*. CRP (mg/L) was measured as part of routine diagnostics at the Department of Laboratory Medicine of the Medical University of Vienna (https://www.kimcl.at). Since participants were not exclusively included at admission, the highest CRP level before inclusion was used for the analysis.

### Statistical analysis

Data are shown as mean ± SEM or median [25th, 75th percentile] based on data distribution. Normal distribution was assessed using the Shapiro–Wilk test. Continuous, normally distributed data (i.e., age in Table 1) were analyzed using a one-way ANOVA. Categorical variables were compared using a Fisher’s exact test. The MxA expression and the serum CRP levels of the different groups were compared using a Student’s t test or a Kruskal–Wallis test with Dunn’s correction as appropriate. A receiver operating characteristic (ROC) curve analysis was performed to assess sensitivity (Se), specificity (Sp), positive likelihood ratio (LR +) and negative likelihood ratio (LR-) of MxA and MxA/CRP ratio to differentiate between viral and bacterial infections. A *p*-value < 0.05 was considered as statistically significant. Data analysis was performed using GraphPad Prism version 9.1.2 for macOS (GraphPad Software, San Diego, California USA, www.graphpad.com) and IBM SPSS Statistics (IBM Corp. Released 2015. IBM SPSS Statistics for Macinstosh, version 23.0.0.2 Armonk, NY: IBM Corp.).

**Table 1:** Demographic and clinical characteristics

Characteristics	All patients (*n* = 61)	Viral infections (*n* = 26)	Bacterial infections (*n* = 26)	Co-infections (*n* = 9)	*P*
Age, y, median (range)	63 (19–88)	62 (21–85)	65.5 (19–88)	63 (54–83)	0.702
Female sex, *n* (%)	17 (27.9)	9 (34.6)	6 (23.1)	2 (22.2)	0.682
Microbiologically confirmed diagnosis, *n* (%)	45 (73.8)	25 (96.2)	18 (69.2)	2 (22.2)^b^	< 0.001
Hospitalized, *n* (%)	54 (88.5)	21 (80.8)	24 (92.3)	9 (100)	0.342
Antibiotic therapy at time of inclusion, *n* (%)	31 (51.0)	0 (0)	24 (96.0)^a^	7 (77.8)	< 0.001
Immunosuppression, *n* (%)	16 (26.2)	4 (15.4)	9 (34.6)	3 (33.3)	0.270
Duration of symptoms > 3 days, *n* (%)	29 (47.5)	8 (30.8)	19 (73.1)	2 (22.2)	0.003

Statistically significance was determined using a one-way ANOVA for “age” and a Fisher’s exact test for all other, categorial variables

^a^record missing in one patient, percentages based on *n* = 25

^b^a virus was detected in all patients with co-infections, bacterial pathogens were detected in 2 patients

## Results

### Patient characteristics

We included 65 patients with clinical signs and symptoms suggestive of an infectious disease (Fig. 1). Blood samples from 3 patients were used to establish the method, and the results were therefore not included in the analysis. One patient was diagnosed with hemophagocytic lymphohistiocytosis and excluded due to possible immunological influences on MxA expression independent of infection. Tables 1 and 2 illustrate the characteristics of the remaining 61 patients included in the analysis. In 25 confirmed viral infections, influenza A was detected in 21 patients, RSV in 3 patients, rotavirus in 1 patient. In one patient classified as virus-infected, no pathogen was detected and diagnosis was made on a clinical basis according to the diagnostic algorithm. Among the patients with bacterial infections, pathogen detection succeeded in 18 (2 *Streptococcus pneumoniae*, 1 *Streptococcus intermedius*, 1 *Streptococcus pyogenes*, 2 *Staphylococcus aureus*, 1 *Hemophilus influenzae*, 1 *Clostridium difficile*, 1 *Campylobacter jejuni*, 1 *Enterococcus faecalis*, 5 *Escherichia coli*, 1 *Klebsiella variicola* 1 *Streptococcus gallolyticus*, 1 *Bartonella quintana*). Of 9 patients with a viral-bacterial co-infection, a viral pathogen was detected in all patients (5 influenza A, 4 RSV) and a bacterial pathogen in 2 patients (1 *S. pneumoniae*, 1 *E. coli*). Six patients suffered from viral-bacterial pneumonia, one patient from influenza A and a concomitant urinary tract infection and one patient from pneumonia and a urinary tract infection.

**Table 2:** Clinical characteristics of patients with viral or bacterial infections

Type of infection	All patients (*n* = 52)	Viral infections (*n* = 26)	Bacterial infections (*n* = 26)
Respiratory tract infection, *n* (%)	29 (55.7)	22 (84.6)	7 (26.9)^a^
Febrile infection, *n* (%)	3 (5.7)	3 (11.5)	0 (0)
Gastrointestinal infection, *n* (%)	5 (9.6)	1 (3.8)	4 (15.4)
Urinary tract infection, *n* (%)	8 (15.3)	0 (0)	8 (30.8)
Other infections	7 (13.5)	0 (0)	7 (26.9)

^a^*p* < 0.001 compared to viral infections using a Fisher’s exact test

Patients with co-infections had more than one leading symptom and are thus not included in this table

### MxA alone or in combination with CRP provides a useful parameter to distinguish between viral and bacterial infections

**Fig. 3:**
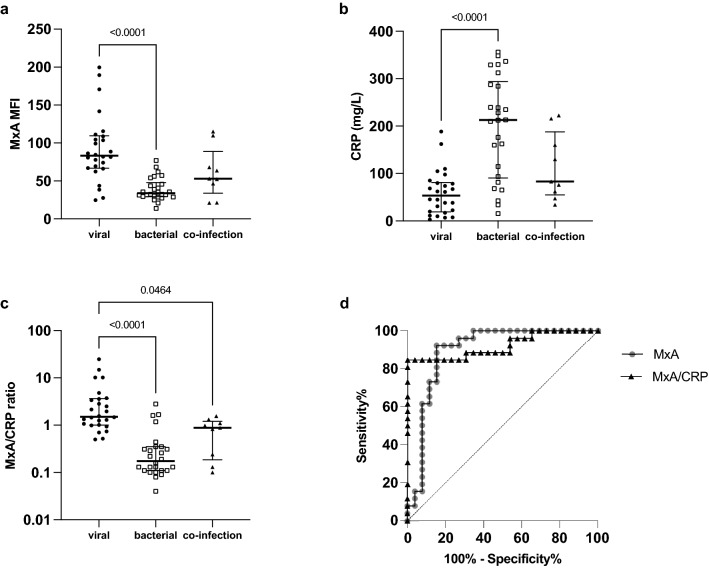
MxA expression in monocytes and CRP levels reliably distinguished between viral and bacterial diseases. **a** Monocyte MxA expression, **b** serum CRP levels and **c** MxA/CRP ratio in patients with viral, bacterial or co-infections. **d** ROC analysis of MxA and MxA/CRP for the differentiation of patients with viral infections and patients with bacterial infections. Data shown as median ± IQR, a Kruskal–Wallis test and a Dunn’s post hoc test were used for comparison

Release of type I/III IFN upon viral infection leads to an upregulation of MxA in infected and uninfected neighboring cells [16]. MxA expression in monocytes after IFN stimulation is higher than in lymphocytes [17]. Therefore, we performed a flow cytometry analysis of monocyte MxA expression in patients with confirmed or clinically diagnosed viral and bacterial diseases. Monocyte MxA expression was considerably higher in patients with viral infections than in patients with bacterial disease (83.3 [66.8, 109.4] vs. 33.8 [29.3, 47.8]; *p* < 0.0001; Fig. 3a). It should be emphasized that monocyte MxA expression was not significantly lower in patients with viral-bacterial co-infections compared to pure viral infections, pointing to an important limitation of the parameter (MFI 53.1 [33.9, 88.9] vs. 83.3 [66.8, 109.4], *p* = 0.19). The AUC of MxA for differentiating between viral and bacterial diseases was 0.9. At a threshold of 62.2, MxA achieved a Se, Sp, LR + and LR- of 92.3% [95% CI 75.9–98.6], 84.6 [95% CI 66.5–93.9], 6.00 [95% CI 2.3–16.0], 0.09 [95% CI 0–0.4], respectively. When only the microbiologically confirmed viral and bacterial infections were used for the analysis, a similar result was obtained with an AUC of 0.88 [95% CI 0.77–0.99]. Se, Sp, LR + and LR- were 88.9% [95% CI 67.2–98.03], 84% [95% CI 65.4–93.6], 5.56 [95% CI 1.9–15.3] and 0.13 [95% CI 0.0–0.5] at the same threshold. CRP levels showed an inverse pattern with significantly higher levels in patients with bacterial infections compared to those with viral infections (21.3 [9.1, 29.4] vs. 5.3 [1.9, 8.1] mg/dl, *p* < 0.0001; Fig. 3b). Previous studies have reported that a combination of MxA and CRP would improve the diagnostic value of MxA [8]. The MxA/CRP ratio was significantly higher in patients with viral infections compared to bacterial infections (15.1 [10.1, 36.3] vs. 1.8 [1.1, 3.5]; *p* < 0.0001) as well as viral-bacterial co-infections (8.8 [1.9, 12.1]; Fig. 3c). Determination of the MxA/CRP ratio further improved diagnostic performance with an AUC of 0.92 [95% CI 0.84–1; Fig. 3d]. At a cutoff value of 4.7, the MxA/CRP ratio achieved a Se, Sp and a LR- of 84.6% [95% CI 66.5–93.9%], 100% [95% CI 87.1–100%] and 0.15% [95% CI 0.1–0.4] (Fig. 3d) for differentiating viral and bacterial infections.

### Subgroup analysis

**Fig. 4:**
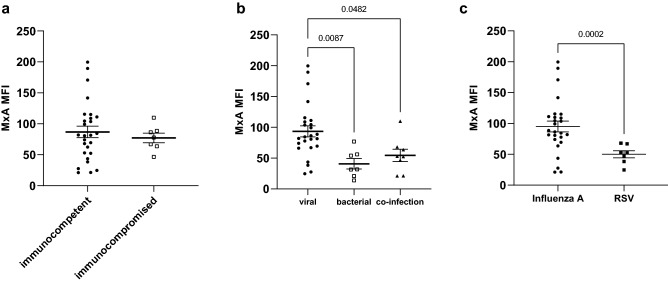
Subgroup analysis **a** Monocyte MxA expression in immunocompetent and immunocompromised patients with viral infections or co-infections. **b** Monocyte MxA expression in patients with fever and/or respiratory symptoms. **c** Monocyte MxA expression in patients infected with influenza A and RSV. Data are shown as mean ± SEM. For comparison, a one-way ANOVA coupled with a post hoc Tukey was used in** b** and a Student’s t test in **c**

We specifically addressed the diagnostic value of MxA in (a) immunocompromised patients and (b) patients with respiratory or febrile symptoms as diagnosing viral infections is especially challenging in these patients. A viral pathogen was detected in seven immunocompromised patients (5 influenza A, 2 RSV). Six patients received immunosuppressive therapy due to solid organ transplantation and one because of graft-versus-host disease after allogeneic bone marrow transplantation. MxA expression was equally induced in these patients compared to immunocompetent patients (MFI 86.9 ± 48.3 vs. 77.1 ± 20.5, *p* = 0.43; Fig. 4a) suggesting that immunosuppressive therapy does not influence monocyte MxA expression and its diagnostic utility. Among patients with febrile and/or respiratory infections, MxA expression was significantly higher in viral infections compared with bacterial infections. (Fig. 4b). Finally, we also analyzed MxA expression in RSV compared to influenza A infections, because previous reports in children suggested that the underlying pathogen might influence MxA induction [10]. Indeed, RSV-infected patients had lower monocyte MxA expression in adult patients compared to influenza A (95.1 ± 45.0 vs. 50.0 ± 15.3 Fig. 4c) which points to a potential diagnostic gap of MxA in clinical routine as RSV represents a common pathogen in adults with respiratory tract infections.

## Discussion

Despite the multitude of diagnostic methods available to modern medicine, the differentiation between viral and bacterial infections often remains a challenge. To close this gap, we measured monocyte MxA expression, which is rapidly upregulated after a viral infection and elevated over a prolonged period [17]. We show that MxA is a reliable marker to distinguish between viral and bacterial infection with high sensitivity. We demonstrate that especially the combination with CRP further improves the diagnostic value of MxA. Our study confirms previous observations [8–10, 18] but also points to important limitations of this biomarker, such as the presence of viral-bacterial co-infections. Furthermore, we show that MxA expression is also useful in immunocompromised adults.

Viral-bacterial co-infections occur frequently and special attention should be paid when interpreting MxA alone in these patients. Although its expression in patients with viral-bacterial co-infections was lower, it did not differ significantly (*p* = 0.19) compared to patients with isolated viral infections in our study. Thus, elevated MxA levels should not be misinterpreted as a pure viral infection leading to a delayed antibiotic therapy. Although a bacterial pathogen could not be identified in some patients with co-infection and thus these patients were classified according to clinical criteria, it is likely that MxA expression is elevated in all viral-bacterial co-infections. Withholding antibiotic treatment in these patients without screening for bacterial infections likely results in an unfavorable outcome. Several studies therefore supported the combination of MxA with a biomarker specific for bacterial infections, such as CRP [8]. Indeed, the MxA/CRP ratio differed significantly between viral infections and co-infections and allowed a more reliable differentiation between viral and bacterial infections in our study.

Toivonen et al. [10] reported that induction of MxA expression in children with respiratory infections depends on the causative virus. While influenza, RSV, coronaviruses and parainfluenza viruses trigger a strong MxA response in symptomatic children with respiratory infections, MxA expression during symptomatic rhinovirus and human bocavirus infections appeared to be lower [10]. In contrast to children, we observed that the MxA response to influenza A virus is higher compared to RSV. Disease severity in viral infections is in part determined by the immune response of the host [19], and the course of RSV infections in adults is usually milder than the flu. Therefore, we hypothesize that the differences in MxA expression are mainly due to the stronger immune response and relate to the severity of the disease rather than the underlying virus. This hypothesis is consistent with the observation that patients with severe courses of COVID-19 have higher levels of MxA [20].

Another important consideration that should be taken into account when interpreting MxA expression is the immune status of the patient. Type I interferon-induced proteins, including MxA, have been used as biomarkers in clinical studies to monitor disease activity and treatment response in patients with autoimmune diseases [21, 22]. Thus, false-positive results can be anticipated in these patients. On the other hand, whether MxA is also a reliable marker in immunocompromised patients has been insufficiently investigated so far. These patients are at high risk for infectious diseases, and differentiating between viral and bacterial is even more challenging due to atypical presentation. Although it is mechanistically conceivable that immunosuppressive therapy impairs the type I interferon immune response, previous studies demonstrated that MxA is a reliable marker for viral infections in immunocompromised children after allogenic stem cell transplantation or chemotherapy [23, 24]. A recent report showed that MxA is also valuable for identifying SARS-CoV2-infected patients, regardless of their immune status [25]. In accordance with these results, we observed no difference in monocyte MxA expression in immunocompromised compared to immunocompetent adults with viral infections.

Our study has some limitations. First, MxA was not measured on the day of hospital admission. However, due to the relatively long half-life of MxA in blood monocytes [17] the delayed measurement of MxA in our cohort probably even underestimates the diagnostic performance of MxA. Second, we used FACS to quantify MxA expression in monocytes, which is a highly accurate and sensitive method, but requires several preparation steps of whole blood samples, laboratory access and experienced personnel limiting its applicability as a rapid point-of-care test. However, recent studies investigated the diagnostic accuracy of FebriDx, a qualitative, rapid immunoassay designed to identify elevated blood MxA and CRP levels and reported comparable results [11–13, 26, 27]. Notably, FebriDx also reliably identified patients with Covid-19 and could be a valuable tool for triage of patients with infectious disease in the future [28–30].

In conclusion, this study demonstrates that MxA is a valuable biomarker to readily detect viral infections. The combination of MxA with a common inflammatory biomarker, such as CRP, has the potential to reduce unnecessary antibiotic prescription.

## Data Availability

Anonymized data are available on request.
